# Diabetes mellitus and tuberculosis facts and controversies

**DOI:** 10.1186/2251-6581-12-58

**Published:** 2013-12-20

**Authors:** Parvaneh Baghaei, Majid Marjani, Pedram Javanmard, Payam Tabarsi, Mohammad Reza Masjedi

**Affiliations:** 1 Clinical Tuberculosis and Epidemiology Research Center, National Research Institute of Tuberculosis and Lung Diseases (NRITLD), Shahid Beheshti University of Medical Sciences, Tehran, Iran; 2 Department of Internal Medicine, SUNY Stony Brook University, Stony Brook, NY USA; 3 Chronic Respiratory Diseases Research Center, National Research Institute of Tuberculosis and Lung Diseases (NRITLD), Shahid Beheshti University of Medical Sciences, Tehran, Iran

**Keywords:** Tuberculosis, Diabetes mellitus, Chronic disease

## Abstract

Tuberculosis (TB) and diabetes mellitus (DM) are both important health issues. A bidirectional association between them has been demonstrated by many researchers. The link of DM and TB is more prominent in developing countries where TB is endemic and the burden of diabetes mellitus is increasing. The association between diabetes and tuberculosis may be the next challenge for global tuberculosis control worldwide. Proper planning and collaboration are necessary to reduce the dual burden of diabetes and TB. One model similar to the TB-HIV program for prevention, screening and treatment of both diseases can be the best approach. In this paper, we review existing data and discuss the matters of controversy that would be helpful for determining research priorities in different countries.

## Introduction

The first report of the association between DM and TB was documented by Avicenna (980-1027 AD) over one thousand years ago. Since that time, the relationship between diabetes mellitus (DM) and tuberculosis (TB), and the nature of their interaction with regards to co-morbidity are largely suggested by numerous epidemiological studies. In the early 20^th^ century, the effect of DM on TB was large concern of investigators, but this was somewhat neglected in the second half of the 20^th^ century with the emergence proper treatment for both diseases [1, 2]. In recent decades, with the increasing prevalence of TB, particularly Multi Drug Resistant TB (MDR-TB), and DM cases in the world, the relationship is re-emerging as a significant public health problem. The link of DM and TB is more prominent in developing countries where TB is endemic and the prevalence of DM is rising.

Although infection with Human Immunodeficiency Virus (HIV) is considered as the most potent risk factor for TB, the high prevalence of DM in the world and its effect on TB burden is greater than HIV infection in many studies [3]. In addition, TB affects DM in many aspects.

Although the definite pathophysiological mechanism of the effect of DM as a predisposing risk factor for TB is unknown, some hypotheses are suggested: depressed cellular immunity, dysfunction of alveolar macrophages, low levels of interferon gamma, pulmonary microangiopathy, and micronutrient deficiency [4, 5].

Few studies in lower income countries have explored this relationship in light of growing DM prevalence in the developing world. Furthermore, the focus of most studies has been to assess the risk of TB in DM patients. In this paper, we reviewed existing data and discussed the matters of controversy that will be helpful for determining priorities of research in different countries.

### Epidemiology of tuberculosis and diabetes mellitus

The prevalence of TB has been rising in recent years globally. It is estimated that in 2010 there were 8.8 million (range: 8.5-9.2 million) new cases of TB. On the other hand, TB is the cause of death for approximately two million people every year [6–8]. The prevalence and incidence of TB in Iran is estimated to be 23 (8.2-40) and 17 (14-21) per 100/000 population respectively in 2010 [9].

Aging, changes in life style, socioeconomic factors, and population growth have lead to an increased prevalence of DM, particularly, type 2 DM. The total number of diabetic people worldwide is predicted to rise from 285 million in 2010, accounting for 3.5 million deaths, to 439 million in 2030 [3, 10, 11]. Up to 80% of patients with DM live in low income and developing countries [12]. Asia is the epicenter of the growing burden of DM [10] and the largest contribution is from India and China [13].

In one nationally representative report from Iran, the prevalence of DM among adults, 25–64 years old, was 7.7% (around 2 million), among whom one half were unaware of their disease. Additionally, 16.8% or 4.4 million of Iranian adults were estimated to have impaired fasting glucose [14].

Worldwide, 70% of diabetics live in TB endemic countries. In the 22 countries with the highest burden of TB, the prevalence of DM in the general population ranges from 2% to 9% [15], and eight of the ten countries with the highest incidence of DM are also classified as high burden countries for TB by the World Health Organization (WHO) [2]. Indonesia, with the third highest burden of TB in the world, has the fourth highest number of diabetics [16]. China, India, Peru and Russia are other countries that need to be given particular attention [17].

Notably, pulmonary TB is the ninth most frequent complication of DM [18] and due to a rising prevalence of DM, the relative contribution of DM to the TB epidemic is increasing [3, 10].

### Diabetes mellitus as a risk factor for tuberculosis

#### Latent infection

The pathophysiology of tuberculosis is complex. Acquisition of the infection is primarily dependent on exogenous factors; however, reactivation of disease is largely under the influence of immune sufficiency [19]. In spite of frequent studies about the link between DM and active tuberculosis, the effect of DM on the frequency of latent TB has been less investigated. The few existing reports about a higher prevalence of latent TB infection among diabetics have been confounded by an absence of control groups [20–22]. Results of one study showed the reaction to purified protein derivate (PPD) is significantly correlated to the degree of hyperglycemia [23]. In other studies, the prevalence of TB infection was not affected by the presence of diabetes [4, 24, 25], or its effect was removed after adjusting for other variables [26]. Therefore, it appears that diabetic patients are not at greater risk for infection with *M. tuberculosis*.

#### Active disease

The frequency of DM among active cases of tuberculosis was 5.6%, 7.3% and 14.8% in studies from India, Turkey and Indonesia, respectively. In 35% to 61% of these patients, DM was diagnosed for the first time after detection of TB [27–29]. Furthermore, impaired glucose tolerance is common [30]. Some suggested that reversible glucose intolerance is not specific for tuberculosis and may occur in the setting of any infection such as pneumonia, [31] but many studies have confirmed a special correlation between DM and active TB.

In 10 case control studies, the pooled odds ratio of TB among DM cases was 2.2 (ranged from 1.16 to 7.81) and in 4 cohort studies pooled relative risk was 2.52 (95% CI: 1.53 to 4.03) [5, 32]. The degree of this effect can be influenced by factors such as age, DM type, severity of DM, prevalence of TB in the region, and ethnicity.

The relation between DM and TB is more prominent in younger people [33]. It seems that patients with type 1 DM are more susceptible than who have type 2 DM. This higher susceptibility may be related to a longer duration of disease or could be due to the fact that control of hyperglycemia is more difficult among type 1 [3, 34]. Additionally, the risk of TB is higher among patients who are using insulin [35], particularly, those who need higher doses of Insulin [36, 37]. Poor glycemic control has been significantly associated with the occurrence of TB [38]. In one study, there was a correlation between active TB and the level of glycosylated hemoglobin (HbA1c) (hazard ratio 1.39, 95% CI: 1.18-1.63 per unit increase) [4].

In populations with a higher incidence of TB, DM is a more important risk factor [33]. DM accounts for a small proportion of TB cases in settings such as Australia with a low incidence of TB [35]. This number was 14.8% in India and 25% in a Mexican setting [39]. Therefore, population attributed risk for TB from DM is dependent upon DM prevalence.

Some authors have suggested that ethnicity may influence the effect of DM on TB. Influence is greater among Hispanic and non-north American populations [19, 33].

Overall, the risk of tuberculosis attributed to diabetes is 25% [27]. At an individual level, Acquired immunodeficiency syndrome (AIDS) is a more potent risk factor for TB in comparison to DM, but due to the high frequency of DM, its effect on the TB burden is equal or even greater than AIDS. In communities with a high burden of HIV infection, the effect of DM may be masked by HIV [2].

Some have suggested that a higher frequency of TB among patients with DM may be related to more frequent contact with health care settings and that transmission of disease is more probable in these settings. Adjustment for contact history as a possible confounding factor, however, did not reduce the strength of the association [40].

Both forms of active TB, primary and reactivated, are equally frequent among DM patients [27, 41].

In summary, data from case control and cohort studies have shown that DM is a risk factor for active TB regardless of study design, incidence of TB in the community, or the place of study [33].

### Effect of diabetes on clinical characteristics of tuberculosis

Diabetic TB patients are usually older than those without DM. This may be due to an association of type 2 DM with older age. Some have reported no difference in term of gender but some reported higher frequency among men [3, 42].

Some symptoms of DM and TB are similar: weight loss and fatigue are common to both [43]. Compared to non-DM patients, TB patients with DM usually have a higher body weight, [3] although some have reported weight loss as being more common among DM cases [28].

A few studies have shown that the clinical characteristics of TB do not differ among diabetic and non-diabetic patients [3, 29, 41, 42, 44–46]. In one study, diabetic TB patients had more symptoms but did not have a more severe form of TB [28].

Extra-pulmonary involvement has been reported to be less common among diabetic TB patients than in non-diabetics [3].

Webb and colleagues showed a higher mean HbA1c among TB-DM in comparison to DM without TB [4], but other studies have shown no difference in the HbA1c among diabetics with and without TB [28, 44].

With regards to the rate of positive smears at the time of diagnosis, results are conflicting. Although some authors reported a higher frequency of negative sputum smears among TB DM cases [28], others found DM as an independent risk factor for numerous acid fast bacilli on the sputum smear examination [29, 42] and some showed no association between DM and patients’ bacteriology results [47]. Conflicting results might be due to the control status of DM [48].

### Effect of diabetes on radiologic manifestations of tuberculosis

There have been conflicting findings regarding the effect of DM on the radiologic characteristics of pulmonary tuberculosis [3, 12]. Concerning the distribution of pulmonary involvement, some studies did not find any difference between DM and non DM cases [28, 44, 49–51]. However, there were other studies that showed a higher incidence of lower lobe involvement among DM TB cases [52–54]. Also, there was no significant difference in the frequency of pleural effusions or isolated pleural TB between patients with and without DM [44, 50].

Although some reported the opposite [28, 46, 49, 51, 55, 56], it seems that cavitary lesions are more common among diabetic patients [29, 41, 44, 53, 57, 58], especially cavitary nodular lesions [44]. Some have suggested this difference may be apparent among uncontrolled DM cases (HbA1c ≥ 7) [48, 59]. Another factor related to the frequency of cavitary lesions was insulin dependency [47]. Furthermore, one study showed an association between lower lung field involvement and female gender or age greater than 40 years [47]. Also, it has been suggested that severe pulmonary involvement in DM patients may actually be related to smoking status and not DM alone [50].

Results of one study of pulmonary TB CT findings showed a high prevalence of non segmental distribution (30%) and multiple small cavities among diabetic patients. However, unusual localization such as lower lobe lesions, involvement of the anterior segment of the upper lobes or right middle lobe, was similar between DM and non DM cases [60].

Of note, differences in patient selection and the definition of DM have to be considered when it comes to some of these discrepancies between different studies [3].

### Effect of diabetes on treatment response of tuberculosis

#### Drug reactions

Diabetes can lead to impaired renal function and an increased risk of drug toxicities. Also, DM is reported as a predictor of drug induced liver injury (DILI) [61]. Hepatic toxicity due to anti tuberculosis drugs may be increased [10, 32] although we didn’t find any relation between DM and DILI in our setting [62].

#### Sputum conversion

Current literature on the effect of DM on sputum bacteriological conversion is very conflicting. Some studies did not show DM to be an independent risk factor associated with increased time to sputum conversion [42] or any relation between DM and sputum conversion rate at the end of 2^nd^ month [12, 28, 42, 63, 64]. On the other hand, there are studies that showed a trend toward increased time to sputum conversion [1, 12, 63, 65]. In one study, uncontrolled DM (HbA1c ≥ 7) was a significant risk factor for positive sputum culture after two months [48].

#### Outcome of TB treatment

Some studies did not show any relation between DM and the outcome of TB treatment [41, 42, 44, 48, 63]. However, DM may have a negative impact on the outcome of TB treatment: higher failure rates [1, 41, 66, 67], higher rates of all-cause mortality [68–70], and death specifically related to TB [71]. In one study, after adjusting for other factors, the chance of death was over six times higher in patients with diabetes [63]. Another study showed an adjusted odds ratio of 7.65 for treatment failure among DM in comparison to non DM tuberculosis patients after removing the effect of covariants such as non-compliance and drug resistance [28]. Definitive causes of death were not reported in most of these studies, therefore, it is not clear whether more severe forms of TB in diabetic patients were responsible, or perhaps it was other comorbidities attributable to DM [12].

Some explanations for worse outcome are higher rates of drug resistance, impaired cellular immunity, delay in sputum conversion, and lower plasma levels of anti TB drugs; the last may be explained by increased weight of DM patients or excess weight gain during TB treatment without an accurate adjustment of drug dosing in the later phase of treatment [3, 72].

Reports have been varied with regards to the effect of DM on the relapse rate of TB. Some have reported a higher incidence of relapse in diabetics [66, 67] and others reported no difference [42, 64, 73]. Also, there is no evidence that DM increases the risk of relapse caused by drug resistant strains [32].

In conclusion, studies examining the effect of DM on treatment outcome are difficult to compare, and few of them have used bacteriological endpoints [3]. Currently, there is not sufficient evidence to recommend alternative anti tuberculosis regimen for diabetics. Consequently, treatment of TB is similar between diabetics and non-diabetics [32].

#### Anti tuberculosis plasma concentration

Patients who have DM may have lower plasma concentrations of anti TB drugs, particularly rifampin [65, 74]. In one study, the mean exposure (AUC_0-6h_) to rifampin was 53% lower in TB DM patients than age and sex matched TB patients without DM in the continuous phase. This effect was associated with the severity of hyperglycemia. Additionally, maximum concentration (C_max_) of rifampin was lower among diabetics. No difference was found between the time necessary for the drug to reach the maximum concentration (T_max_) [75]. Surprisingly, there were no differences in the pharmacokinetics of rifampin, pyrazinamide, and ethambutol in the intensive phase [54]. The exact mechanism of lower plasma drug level is not defined. A decrease in gastric hydrochloric acid secretion [74] and impaired drug absorption, even in the absence of clinical gastroparesis may be the reasons [49]. One study showed no effect on drug levels [76].

#### Drug resistance to anti tuberculosis drugs

Multi Drug Resistant (MDR) tuberculosis (concomitant resistant to isoniazid and rifampin) is an increasing challenge against the control of TB throughout the world. Some studies reported no relationship between DM and MDR TB [42, 48, 58, 77, 78]. On the other hand, many authors have found an increased risk of MDR TB among diabetics [1, 73], ranging from 2.1 to 8.8 times more common [49, 79, 80]. Also, in one study diabetic patients frequently relapsed with resistant strains [64].

There is no proven explanation about the impact of DM on drug resistancy in tuberculosis. One mentioned hypothesis is related to the katG gene that is involved in the protection of the mycobacterium against oxidative damage and also encodes an enzyme which transforms isoniazid to the active form. In type 2 diabetics, production of reactive oxygen species may be impaired, so strains with katG mutations may be better able to survive [81].

### Effect of tuberculosis on diabetes mellitus

Glucose intolerance has been reported among 16.5% to 49% of patients with active TB. In one study, 56.6% of cases with glucose intolerance at the time of diagnosis had normal glucose levels after treatment of the TB, a phenomena called “transient hyperglycemia” [59, 82]. Additionally, it must be noted that control of hyperglycemia is more difficult during the active phase of tuberculosis and many patients require insulin for control of hyperglycemia [83].

Although a definite cause of hyperglycemia associated with TB had not been identified, some probable mechanisms have been suggested [39]. Inflammation caused by cytokines such as IL6 and TNFα in response to TB infection may cause an increase in insulin resistance and decreased insulin production, thereby leading to hyperglycemia [84].

Additionally, Isoniazid and rifampin have hyperglycemic effects. Also pyrazinamide may result in difficult control of DM [39, 83, 85–87]. Rifampin induces metabolism and decreases blood level of sulfonylureas, leading to hyperglycemia [3]. The maximum effect of this is seen about one week after starting and disappears two weeks after discontinuing rifampin [88]. Rifampin doesn’t affect the metabolism of metformin or insulin [3].

### Tuberculosis screening among diabetics

Some believe that similar to other populations susceptible to TB, (i.e. HIV-infected individuals, gold miners, and prisoners in developing countries) screening for active TB among diabetics could improve case detection and could consequently lead to earlier therapy and prevent transmission of disease [36].

The number of diabetics needed to screen to find one extra case of TB is directly related to the local TB prevalence. For example, in settings with a TB prevalence less than 25 per 100,000 persons, at least 1,000 diabetic persons have to be screened to find one extra case of TB. When the prevalence is greater, the number needed to screen to find one additional case of TB ranges from 4 to 442. Therefore, the yield of screening increases with the prevalence of TB in the region [36]. Surveillance is crucial in deciding which form of planning is suitable in settings with medium to high TB burden with an estimated TB prevalence exceeding 100 in 100,000 population [32].

The best method for screening of TB is not yet defined. One possible strategy consists of performing chest X-rays at the time of DM diagnosis, and at regular intervals thereafter However, less specific methods such as imaging may lead to over diagnosis [10, 36].

It is sensible that any diabetic patient with suspicious symptoms such as cough for more than 2–3 weeks, weight loss, fever, or an abnormal imaging study should be investigated for presence of active TB. Screening is recommended, especially in uncontrolled diabetics and diabetic children with recent TB exposure. There is currently insufficient evidence for more active screening measure [4, 5, 32].

### Prophylaxis for tuberculosis among diabetics

The American Thoracic Society has recommended performing tuberculin skin test (TST) with purified protein derivative (PPD) for all diabetic patients. If the induration is 10 mm or more, prophylactic treatment with isoniazid is recommended for 6 to 12 months, unless the patient has had a history of tuberculosis [89]. Some authors have questioned the actual benefit of this recommendation [90]. Of note, the prevalence of *M. tuberculosis* infection among diabetics is high and the sensitivity of TST may be reduced [4].

Only two studies have investigated the advantage of TB prophylaxis among diabetics. The first was conducted in Germany in the 1950s where post treatment prophylaxis with isoniazid for 6–24 months after completion of a full course of treatment for active TB had been evaluated in diabetic patients. Recurrence rates were lower in the intervention group [91]. In a second study conducted in Russia in the 1960s, administration of an analogue of isoniazid for diabetics lowered the incidence of TB compared to controls by 2 to 3 times [92]. Both studies were problematic due to the absence of randomization and the lack of details regarding the interventions [10]. Therefore, the true effectiveness of chemoprophylaxis in diabetic patients has remained unknown and only through a randomized controlled trial can it be properly addressed. However, conducting of such studies is expensive. Furthermore, the experience with regards to people living with HIV have showed poor patient compliance, despite proven efficacy. Therefore, this research is not high priority because it is unlikely to change current policy and practice [5, 93].

In brief, there is not sufficient evidence to support any preventive therapy for diabetics with latent TB infection. However, preventive therapy may be considered for certain high risk groups such as diabetics who have a close TB contact [5, 32].

### Screening for diabetes among patients with tuberculosis

A wide range of DM prevalence from 1.9% to 35% was reported by screening for DM among patients with TB. The highest values were reported from regions with high prevalence of DM. Many of these patients were newly diagnosed as a result of receiving expanded medical attention related to TB treatment [12, 27, 36, 45, 59].

In particular, type 2 DM is often unrecognized. In two studies from Tanzania and Indonesia, 73% and 61% of diabetics, respectively, were newly diagnosed concurrent with active TB [3, 28, 30]. Screening for DM in patients with TB could improve case detection, early treatment, and prevention of DM complications [36]. Older age, obesity, inactive lifestyle, and family history of DM are risk factors for DM among TB patients [28, 45].

The preferred method for screening of DM among TB cases has not been determined. Measurement of fasting blood glucose (FBG), random blood glucose (RBG) and 2 hour postprandial glucose (2hPG), urine glucose, HbA1c, and performance of glucose tolerance test (GTT) have been suggested [5, 10, 27, 40]. In one study, questions about symptoms of hyperglycemia led to the diagnosis in all of the DM cases [45].

Some authors recommend measurement of 2hPG as the best method. It is easy, cheap, rapid, and reliable. This method is regularly more sensitive than FBG and RBG [32]. In Asian populations, the sensitivity of 2hPG is reportedly higher than FBS and HbA1c [13].

The World Health Organization (WHO) recommends HbA1c as a diagnostic test for DM. However, it is expensive and use of this test alone is still controversial. Urine testing for glucose is insensitive and suboptimal, especially in the early stages of DM [10].

The best time for screening is not yet clear. Some recommend screening for DM later in the disease process, when TB treatment has shown its effect. The reason for this is that, as an infectious disease, TB may transiently elevate blood glucose and an infection related hyperglycemia may result in misclassification as DM [12, 36]. On the other hand, early screening for DM has some benefits including initiation of diabetes treatment, education of patients and correction of hyperglycemia, which potentially could have positive effects on the outcome of TB treatment. Additionally, national TB programs in many countries refer TB patients to peripheral facilities where laboratory investigations are difficult to perform. Therefore, screening for diabetes is recommended at the start of TB treatment [32, 93]. As hyperglycemia may regress after treatment of TB, verification of glucose intolerance after cure of TB is necessary. Even after the return of blood sugar to normal level, subsequent monitoring is necessary, because it has been shown that a history of impaired fasting glucose is a strong predictor of subsequent diabetes [45, 94]. Due to these reasons, some recommend screening both at the time of diagnosis of TB and three months later after initiating treatment [5].

The number of TB patients needed to screen for detection of one extra case of DM ranged from 4 to 54 in several studies [36]. As a result, screening of DM among TB cases is more cost-effective than screening of TB among DM cases.

### Unresolved issues

Many critical questions remained unanswered. Well designed studies are necessary to determine the optimal time and method of screening for DM in TB patients. Also, the best screening algorithm for diagnosis of TB among diabetics is unknown. The role of newer tools for detection of M. *tuberculosis* infection, such as commercial IFN-γ release assays has not yet been determined. Larger studies with more definite endpoints are needed to evaluate the effect of DM on the outcome of TB treatment. The effectiveness of TB preventive therapy may only be answered through a randomized controlled trial.

## Conclusion

The burden of diabetes mellitus is increasing worldwide. The association between diabetes and tuberculosis is the next challenge for global tuberculosis control. Improved understanding of the bidirectional relationship of the two diseases is necessary for proper planning and collaboration to reduce the dual burden of diabetes and TB. In people with TB, it may be appropriate to actively screen for DM. Prevention, screening, and treatment of both diseases together is more effective. Perhaps, a model similar to the TB-HIV program may be the best approach.

## Abbreviations

DM: Diabetes mellitus
TB: Tuberculosis
MDR: Multi Drug Resistant tuberculosis
HIV: Human Immunodeficiency Virus
WHO: World Health Organization
PPD: Purified protein derivate
CI: Confidence interval
HbA1c: Hemoglobin A1c
AIDS: Acquired immune deficiency syndrome
CT: Computed tomography
DILI: Drug induced liver injury
AUC 0–6 h: Area under curve 0–6 hour
Cmax: Maximum concentration
Tmax: Time necessary for drug reach to the maximum concentration
TST: Tuberculin skin test
M. tuberculosis: Mycobacterium tuberculosis
FBG: Fasting blood glucose
RBG: Random blood glucose
2hPG: 2-hour plasma glucose
GTT: Glucose tolerance test
IFN-γ: Interferon gamma.
